# Global Trends in Research of NF-*κ*B in Melanoma from 2000 to 2021: A Study of Bibliometric Analysis

**DOI:** 10.1155/2022/3684228

**Published:** 2022-09-10

**Authors:** Jun Wang, Xuan Liao, Xiao Jiang, Hongwei Liu

**Affiliations:** ^1^Department of Plastic Surgery, The First Affiliated Hospital of Jinan University, Guangzhou 510630, China; ^2^Department of Burn and Skin Repair Surgery, Hainan General Hospital, Hainan Affiliated Hospital of Hainan Medical University, Haikou 570311, China; ^3^Key Laboratory of Regenerative Medicine, Ministry of Education, Guangzhou 510630, China

## Abstract

In the pathogenesis of melanoma, NF-*κ*B is a key signaling pathway. Appling bibliometric analysis, we identify the frontiers and hotspots about NF-*κ*B in melanoma, as well as distinguishing features of scientific research and output all over the world during the past 22 years. 2226 publications published from 2000 to 2021 and related information were retrieved based on Science Citation Index Expanded (SCI-expanded) of Web of Science Core Collection (WoSCC). VOSviewer and Citespace were used to analyze bibliometric indicators and visualize the hotspots and research trend of studies on NF-*κ*B in melanoma. The results indicated that despite fluctuations, the number of publications (Np) related to the research of NF-*κ*B in melanoma per year increased over the past 22 years. The USA had the most publications. H-index and the number of citations (Nc) of the USA were also in the first place. PloS One was the most productive journal, and League of European Research Universities (LERU) was the most productive affiliation. Recently, the keywords “NF-kappa-b,” “melanoma,” “apoptosis,” “expression,” “activation,” “cancer,” and “metastasis” appeared most frequently. Our study suggested that articles associated with NF-*κ*B in melanoma tend to increase. In this field, the USA was an influential country and a big producer. Most publications focused on clinical and basic research in the past 22 years, and keywords “tumor necrosis factor” and “trail induced apoptosis” had the highest burst strength.

## 1. Introduction

Melanoma refers to a tumor derived from melanocytes with a high degree of malignancy. Abnormal and excessive proliferation of melanocytes in neural crest is the main cause of its pathogenesis [1]. The incidence rate of melanoma is increasing worldwide. 95% of melanoma patients died of brain metastasis at the end of the disease [2]. The annual mortality of patients with melanoma is higher than 3.5%, and the 5-year survival rate of patients with metastatic melanoma is about 15%∼20% [3]. About 10% of melanoma cases are diagnosed as advanced, which has metastasized and cannot be removed [4]. Inhibiting the invasion and migration of melanoma is critical to ameliorate the survival rate of melanoma patients, and it is urgent to further explore the molecular mechanism of melanoma and seek a treatment scheme with good curative effect to improve the prognosis of patients. Epidemiological studies show that the incidence rate of melanoma is increasing worldwide. It may be due to the early diagnosis and improvement of health cognition obtained by skin biopsy, thus diagnosing a large number of cases with very thin and marginal cutaneous melanoma [5]. Studies have shown that the joint action of internal factors (such as genetic factors) and external factors (such as ultraviolet light) leads to the pathogenesis of melanoma [4]. Multiple signaling pathways play an important role in the pathogenesis of melanoma [6–8].

As an excellent transcription factor, nuclear factor-kappa B (NF-*κ*B) is a significant role in the invasion and metastasis of many cancers [9]. It also plays a variety of roles in cell survival, differentiation, and proliferation [10]. The NF-*κ*B transcription factor family consists of five different proteins: rela, RelB, c-Rel, P100, and P150 [11]. It is one of the targets of tumor therapy [12]. The activation of NF-*κ*B has been proposed as an event that promotes melanoma tumor progression [13]. Constitutively activated NF-*κ*B signaling pathway plays an important role in melanoma initiation, progression, invasion, metastasis, and resistance to chemotherapy and immunotherapy, and it is the convergence point of dysregulated cellular signaling pathways in melanoma [14]. Shomali et al.'s study suggested that HSP90 may act as a potential therapeutic target in melanoma. However, more studies are needed to determine the exact role of HSP90 and its association with HMG genes [15]. At present, there are mainly immunotherapy, braf/mek inhibitors, and other methods for the treatment of melanoma. In recent years, there are more and more studies on NF-*κ*b in melanoma [16–18]. Professors and scholars continue to make breakthroughs in the field. The emergence of new concepts and the introduction of new technologies are also great challenges for researchers. Therefore, it is necessary to summarize the research progress in this field. Bibliometrics is defined by Pritchard as “the application of mathematical and statistical methods to books and other media.” Historical bibliometrists recognize that adding time and space dimensions to bibliometric analysis can bring new insights into knowledge development and academic records [19]. Bibliometric methods are majorly composed of citation analysis, which is the source of influencing factors. The number of citations of the research center is considered to be high. Therefore, the top ranked and highly cited papers provide evidence and information for research trends and scientific progress in specific fields [20]. Bibliometrics and visual analysis can effectively support information integration to improve the understanding of research activities [21]. Over the recent years, bibliometric indicators are increasingly used to evaluate and manage research activities [22]. Bibliometrics is a method of quantifying the research object, which is often used to determine the law of literature. Unlike the traditional narrative review, which depends on the experience and knowledge of researchers, bibliometric analysis takes science as a knowledge generation system [23]. Nowadays, there are many bibliometric research results, such as metformin [21], tourism [24], artemisinin [25], diabetic foot ulcers [26], and macrophages associated with acute lung injury [27]. Nevertheless, bibliometric research on NF-*κ*B in melanoma is still a blank. Therefore, the purpose of this study is to systematically analyze study of NF-*κ*B in melanoma to make a scientific and comprehensive evaluation of the hotspots and research status in the field.

## 2. Materials and Methods

### 2.1. Data Source and Search Strategy

The publications related to NF-*κ*B in melanoma from 2000 to 2021 were obtained based on the Science Citation Index Expanded (SCI-E) of the Web of Science Core Collection (WOSCC) on April 22, 2022. The retrieval topic was “(TS = (Melanoma)) AND (TS = (NF-kappa B) OR TS = (NF-kB) OR TS = (nuclear transcription factor-*κ*B)),” and only the “review” and “article” written in English were included in this study. 2226 publications meeting the search criteria in total were selected to carry out further analysis.

### 2.2. Analytical Methods

With CiteSpace 5.8.R3 and VOSviewer 1.6.16, data visualization and analysis were carried out. CiteSpace, developed by Professor Chaomei Chen, is a piece of software which can visualize networks among research hotspots and documents as well as citation collaboration [28]. As a software focusing on bibliometrics network, VOSviewer can well analyze literature information, such as keyword cooccurrence, cocitation, and coauthorship. In conclusion, these analytical tools provide an objective and different view of development. The number of publications (Np), the number of citations without self-citations (Nc), and the academic contribution of researchers are evaluated by the H-index, affiliation, country, or journal [27]. In 2005, Hirsch first introduced the H-index. As a comprehensive scoring, it can well assess the importance and wide-ranging impact for cumulative research contributions of scientists [29].

## 3. Results

### 3.1. General Statistics

**Figure 1:**
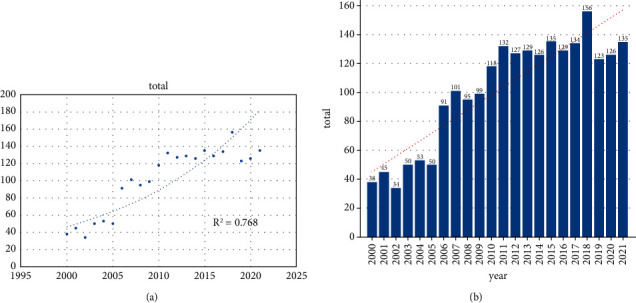
(a) Curve fitting of the total annual growth trend of publications. (b) The number of publications by year over the past 22 years.

There were 2,226 publications about NF-*κ*B in melanoma from the SCIE of WoSCC during 2000–2021. We found that 75 countries/regions participated in the research field, and these publications were from 623 journals, 2,326 institutions, and 12,830 authors. There were 8,894 keywords included in all publications. Publishing types were divided into two categories: there were 1,827 research articles accounting for 82.08% and 399 reviews. The total Nc of all publications was 118,172, and the Nc per publication was 55.05. The H-index for all retrieved publications was 148.  Figure 1(a) shows the fitting curve of the annual trend of the number of papers published. Annual Np is not related to the year of publication. As can be seen from Figure 1(a), the correlation coefficient *R*^2^ was only 0.768. Figures 1(a) and 1(b) show the annual Np associated with NF-*κ*B in melanoma. In summary, despite fluctuations in the number of documents issued over the past 22 years, the number of annual papers rose from 34 in 2002 to 156 in 2017.

### 3.2. Performance of Country/Region

**Figure 2:**
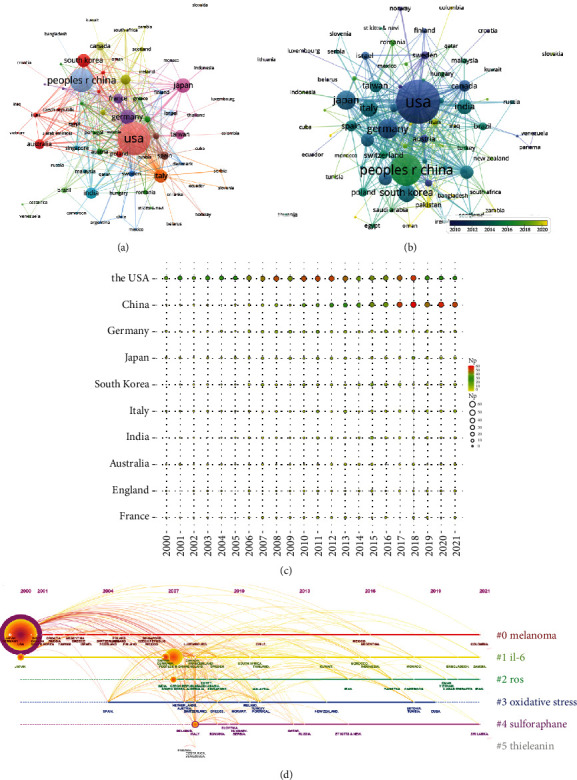
Leading countries. (a) Visual cluster analysis of cooperation among countries. (b) Timeline visualization of cooperation among countries. (c) The number of documents issued by the top ten countries each year. (d) Timeline distribution of the top 6 clusters.

Over 2000–2021, groups from a total of 75 countries/regions published NF-*κ*B in melanoma-related articles. Figures 2(a) and 2(b) show the cooccurrence of all the countries. The top 10 most productive countries are shown in Table 1 and the number of documents issued by these countries each year is shown in Figure 2(c). The United States produced the most NF-*κ*B in melanoma-related articles, which was 847 publications in the studied period. China, Germany, and Japan were the following countries in terms of Np. The United States also showed the highest Nc of 66218 and H-index of 117. Even though China is the second productive country, its Nc was lower than Japan. Visualized timeline of countries is shown in Figure 2(d). There were six clusters including melanoma, IL-6, ROS, oxidative stress, sulforaphane, and thieleanin.

### 3.3. Affiliation and Author Contributions

**Figure 3:**
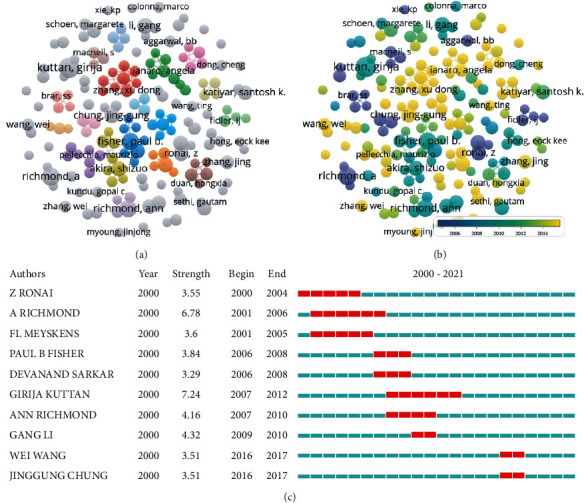
Author analysis. (a) Visual cluster analysis of cooperation among authors. (b) Timeline visualization of cooperation among authors. (c) Representative burst authors with the strongest citation bursts.

**Figure 4:**
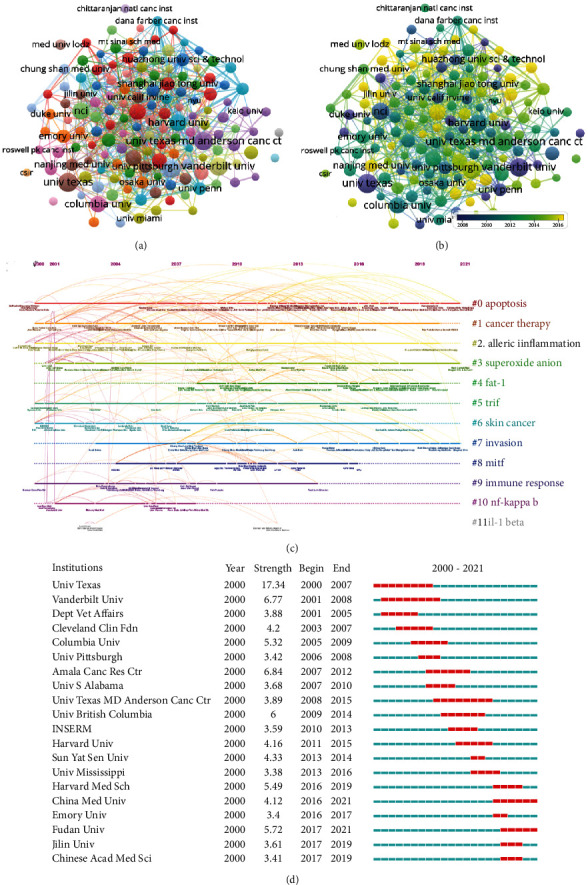
Affiliation analysis. (a) Visual cluster analysis of cooperation among affiliations. (b) Timeline visualization of cooperation among affiliations. (c) Timeline distribution of the top 12 clusters. (d) Representative burst affiliations with the strongest citation bursts.

A total of 2,326 affiliations and 12,830 authors have contributed to the field of NF-*κ*B in melanoma.  Table 2 shows the top 10 affiliations with the highest number of publications. League of European Research Universities (LERU) occupied the first place in Np, and University of Texas System ranked first in Nc and H-index. Although UT MD Anderson Cancer Center ranked 4th in Nc, its H-index was higher than University of California System. To find the most influential researchers in the NF-*κ*B in melanoma field in the last 22 years, according to the H-index and publication number, we ranked the top 10 authors. As the most productive author, Richmond A from Vanderbilt Univ of the United States was with Np of 29 and an H-index of 26. Kuttan G from Amala Canc Res Ctr and Ivanov VN from Columbia University had an H-index of 16 and 14, respectively (Table 3). Author cooccurrence is shown in Figures 3(a) and 3(b), and the top 10 authors with the strongest citation bursts are shown in Figure 3(c). Affiliation cooccurrence is shown in Figures 4(a) and 4(b). Visualized timeline of affiliations is shown in Figure 4(c). There were 12 clusters, and the top 20 authors with the strongest citation bursts are shown in Figure 4(d).

### 3.4. Performance of Journal

The 2,226 publications were published in 623 journals. For the all publications, publications in the top 10 productive journals accounted for 19.23% (Table 4). PloS One ranked first in Np (62). CANCER RESEARCH had the highest H-index (39) and IF (12.701), and ONCOGENE had the highest Nc (5962). 8 journals were from the United States, 1 journal was from England, and 1 journal was from the Netherlands. Impact factor (IF) is a recognized sign to determine the influence of journals based on the frequency of journal articles cited by other scientific publications. Except that PLoS One has a low impact factor or Oncotarget has been removed from science, other journals have high IF (IF > 5).

### 3.5. Hotspot Detection and Burst Analysis

**Figure 5:**
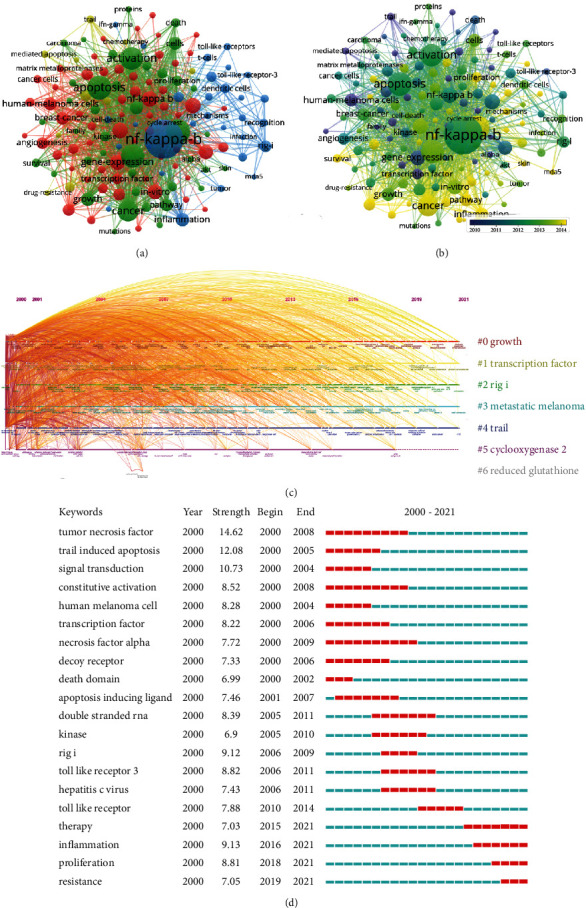
Keyword analysis. (a) Visual cluster analysis of cooccurrence among keywords. (b) Timeline visualization of keywords. (c) Timeline distribution of the top 7 clusters. (d) Representative burst keywords with the strongest citation bursts.

Research hotspots and frontiers in a certain field can be reflected by keywords. The top 20 most frequently keywords were NF-kappa-b (1306), melanoma (502), apoptosis (491), expression (457), activation (436), cancer (372), metastasis (213), NF-kappa b (209), melanoma cells (206), gene-expression (193), in-vitro (182), malignant-melanoma (176), inflammation (154), growth (151), human-melanoma cells (148), cells (146), inhibition (146), breast-cancer (138), down-regulation (123), and pathway (123). Keyword clustering and visualization can be carried out through VOSviewer. Due to occurrence >25, 143 keywords reached the specifications and 4 clusters emerged. As shown in Figure 5(a), each word in the 143 keywords is represented by a circle, and the frequency of occurrence of keywords is represented by the size of the circle. A connection line is generated when at least one of the two keywords connected to a keyword coexists. The different colors represented the 4 clusters: cluster 1 (in red) and cluster 2 (in green) mainly concentrated in mechanism research; cluster 3 (in yellow) focused on inflammation research; cluster 4 (in blue) focused on basic and clinical researches. As shown in Figure 5(b), based on the average publication year (APY), all keywords were divided into different colors with VOSviewer. Keywords in the recent six years were tumor microenvironment (2017.24), inflammasome (2017.03), autophagy (2016.33), and epithelial-mesenchymal transit (2016.11). Compared with Figures 5(a) and 5(b), the research of anti-inflammation for alleviating melanoma was relatively the latest. As shown in Figure 5(c), the top 7 clusters of keywords were “growth,” “transcription factor,” “rig i,” “metastatic melanoma,” “trail,” “cyclooxygenase 2,” and “reduced glutathione.” We found that “tumor necrosis factor” and “trail induced apoptosis” were with the highest burst strength (Figure 5(d)). Studies have shown that TNF-*α* treatment of melanoma can induce consistent dedifferentiation [30]. Furthermore, the latest keywords “therapy,” “inflammation,” “proliferation,” and “resistance” emerged in the last 6 years.

### 3.6. Cocitation Analysis

**Figure 6:**
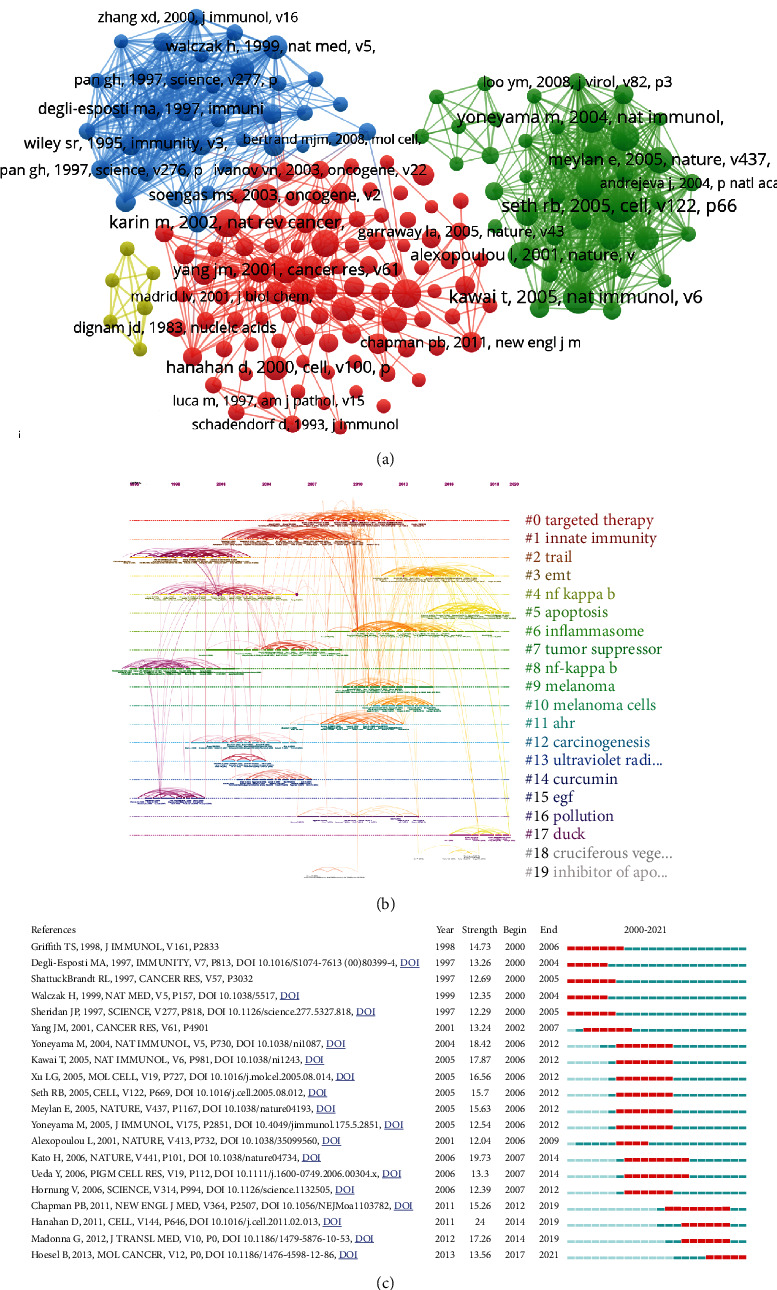
Cocited reference analysis. (a) Visual cluster analysis of cooccurrence among cocited references. (b) Timeline distribution of the top 10 clusters. (c) Representative burst cocited references with the strongest citation bursts.

A cocitation relationship was constituted when two or more articles are simultaneously cited by more than one subsequent publication. The higher the cocitation number, the stronger the cocitation relationship, indicating that these articles have high similarity and will produce a common theme. We performed cluster analysis from the cocitation relationship analysis to further delineate the research frontier and obtain the publications with critical citations from a chronological perspective with CiteSpace and VOSviewer. Due to a large number of cited references, we set the minimum citations per reference as 20. Among the 103304 references which were cited by these publications, we selected 181 references for further analysis (Figure 6(a)). There were 96 references in cluster 1 (in red), which mainly paid attention to drug therapy and mechanism of melanin. Cluster 2 (in green) mainly paid attention to study on various signaling pathways in cancer. Cluster 3 (in blue) focused on study of TNF-related apoptosis inducing ligand (TRAIL) and NF-*κ*B in melanoma. The subject of cluster 4 (in yellow) was therapeutic effect and mechanism of betulinic acid on tumor. Furthermore, a visualized timeline of clusters was carried out (Figure 6(b)). We found that “NF-kappa B” and “EGF” are early fields in the study of NF-*κ*B in melanoma. However, the current hotspots of NF-*κ*B in melanoma are on “EMT,” “apoptosis,” and “duck.” Finally, a reference burst was conducted.  Figure 6(c) shows the top 20 references that possessed the strongest citation bursts and the most representative references in terms of burst strength, burst duration, and burst time. The study of Griffith TS et al. (1998) possesses the highest bursts strength. The latest reference was the paper written by Hoesel B; in his article, he provided an overview that during cancer and inflammation, NF-kappa B and other signaling molecules had the most relevant modes of cooperativity and crosstalk. The top 10 cocited journals are shown in Table 5, and the most frequently cited journal is Cancer Res.

### 3.7. Bibliographic Coupling Analysis

**Figure 7:**
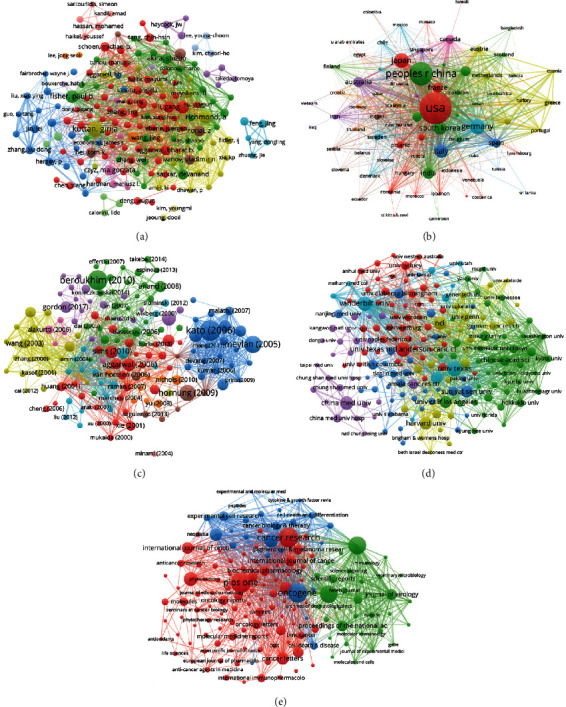
Bibliographic coupling analysis. (a) Author cooccurrence. (b) Country cooccurrence. (c) Publication cooccurrence. (d) Affiliation cooccurrence. (e) Journal cooccurrence.

Bibliographic coupling refers to the phenomenon that two or more documents cite one document at the same time. Ranked by total link strength, top 10 authors in bibliographic coupling analysis were Richmond, A (7139), Fisher, Paul B. (5259), Ivanov, Vn (4723), Akira, Shizuo (4535), Richmond, Ann (4398), Sarkar, Devanand (4001), Ronai, Z. (3571), Zhang, Xu Dong (3178), Amiri, Ki (3150), and Colonna, Marco (3134). The top ten countries were the USA (228698), China (95306), Germany (75474), Italy (53416), Japan (52034), Australia (33843), South Korea (31695), Spain (29333), India (28982), and England (24595). The top 10 publications were Amiri (2005, 282), Almasan (2003, 270), Leblanc (2003, 254), Richmond (2002, 243), Raman (2007, 236), Soengas (2003, 230), Basseres (2006, 212), Cui (2014, 211), Kumar (2006, 209), and Gitlin (2006, 206). The top 10 affiliations were Vanderbilt Univ (19469), Univ Texas (14945) Columbia Univ (10203), Osaka Univ (10070), Univ Texas MD Anderson Canc Ctr (8976), Kyoto Univ (8676), NCI (7604), Harvard Univ (7121), Washington Univ (7079), and Dept Vet Affairs (6892). The top 10 journals were Oncogene (13026), Journal of Immunology (10892), Cancer Research (10041), Journal of Biological Chemistry (9912), Journal of Virology (8377), Clinical Cancer Research (7501), PloS One (6761), Cancer Biology & Therapy (4703), International Journal of Molecular Sciences (4573), and Journal of Investigative Dermatology (4529).  Figure 7 shows the bibliographic coupling analysis.

## 4. Discussion

Melanoma is a form of skin cancer that occurs in areas where there is little exposure to sunlight. Melanoma is mainly found in the back of men and the legs of women. This type of tumor develops from existing moles and it is usually irregular in shape and uneven at the edges. This type of skin cancer is a disease that can lead to fatal results depending on the stage of diagnosis. The five-year survival rate of patients with metastatic melanoma is less than 15%. Almost all organs are the target of metastasis. However, the liver, bones, and brain are most often affected. Local chemotherapy is becoming a promising strategy to avoid the sequelae of traditional treatment. Therefore, it is necessary to implement newly developed alternative therapies, such as the use of liposomes as drug carriers to prevent a variety of skin diseases [31].

We conducted a bibliometrics analysis according to 2226 articles related to NF-*κ*B in melanoma from the SCIE of WoSCC database during 2000–2021 with computational algorithm and multiple literature analysis software. Our work summed up research trends, hotspots, and frontiers for NF-*κ*B in melanoma, and a summary of global research on its impact was obtained. Over the past 22 years, the annual number of publications fluctuates, but on the whole, it shows an upward trend, indicating its rapid development and constant research interest of NF-*κ*B in melanoma. As for the top countries/regions, the USA ranked first in Np, indicating that the USA was a highly productive country on NF-*κ*B in melanoma. In the top 10 authors, six authors came from the USA in the research of NF-*κ*B in melanoma, suggesting that the USA has the most professional researchers in the world, and it explained why the USA developed rapidly in this field over the past 22 years. Compared with China, Japan had a moderately high Nc, although Japan's Np is much lower than that of China. This showed that Chinese scholars and institutions should make more efforts on the quality of papers in this field. Notably, eight of the 10 most productive journals had higher IF. This means that publishing studies on NF-*κ*B in melanoma in high-quality journals is not a challenge.

Keyword analysis shows that the research of inflammation in melanoma is a research hotspot this year. There is increasing evidence that systemic inflammatory response is an important determinant of tumor progression and survival in many malignancies [32]. Several stages occur in the cellular process that transforms normal melanocytes into tumor cells. From benign nevus to mature tumor cells, genetic instability and proinflammatory environment can lead to tumorigenesis and metastasis. In the cellular microenvironment, immune cells and immune-related molecules play a decisive role in the inflammatory environment. Although the typical cell interface studied in tumors is between CTLS and cancer cells, the contribution of other immune cells is now widely recognized. These immune cells set up complex immune responses in cancer, including promoting tumors and promoting cancer progression. In some unpredictable situations, the clinical evolution of melanoma requires additional prognostic markers to identify patients at early stage and high risk of melanoma, thereby contributing to improved clinical surveillance strategies and treatment management. One of these additional biomarkers includes inflammatory immune cell infiltration that may describe local antitumor responses or may trigger protumor pathways [33]. Recent data suggest that secreted inflammatory cytokines play a paracrine role in the tumor microenvironment and also promote tumor growth. IL-1 expression stimulates angiogenesis and promotes tumor growth. During the evolution of melanoma, activated macrophages produce TGF-*β* (transforming growth factor-*β*), TNF-*α* (tumor necrosis factor-*α*), IL-1*α* (interleukin 1*α*), arachidonic acid metabolites, and extracellular proteases, while melanocytes express IL-8 and VEGF-*α* (vascular endothelial growth factor-*α*) and induce angiogenesis [34].

Proliferation is another research hotspot in this field. Studies have shown that dendritic cells (DCS) are active molecules that indirectly resist the proliferation of melanoma cells. In addition, DC maturation, migration, and cross-initiation, as well as their functional interactions with cytotoxic T cells through immune checkpoint receptor ligands, are impaired. Many signals are transmitted by highly proliferating melanoma cells and helper cells as T cells, natural killer cells (NKs), tumor-associated macrophages (TAMs), T-regulatory cells (T-Regs), and myeloid suppressor cells (MDSCs), and endothelial cells contribute to the immunosuppressive environment. Results Phagocytosis of tolerance factors and interleukins (IL), such as IL-6 and IL-10. To highlight the role of immune infiltration in blocking melanoma progression, the composition, density, and distribution of cytotoxic T cells in the surrounding stroma have been described as predictors of response to immunotherapy [35]. In addition, NF-*κ*B is an important pathway for melanoma to proliferate [36]. Punita Dhawan et al. reported that, through inhibitors of IKK, NF-*κ*B could be suppressed and then inhibit the proliferation of melanoma cells [37]. The research of An'an XU suggested that inflammatory cytokines could activate NF-*κ*B and induce the expression of proinflammatory cytokines, so accelerating the development of melanoma [38]. In recent years, although targeted therapy and immunotherapy have completely changed the treatment of metastatic melanoma, most patients have not been cured. Treatment of drug resistance remains a major clinical challenge. Melanoma includes cell subsets with different phenotypes, showing different genetic characteristics, leading to tumor heterogeneity and conducive to therapeutic resistance. Cellular plasticity in melanoma is called phenotypic transformation. Regardless of their genomic classification, melanoma will change from a proliferative and differentiated phenotype to an invasive, dedifferentiated, and usually treatment-resistant state [39]. Advances in the use of targeted therapy and immunotherapy have revolutionized the clinical management of melanoma patients, significantly prolonging their overall and progression-free survival. However, due to gene mutation and epigenetic modification, targeted therapy and immunotherapy are limited, which determines the huge heterogeneity and phenotypic plasticity of melanoma cells. Acquired resistance of melanoma patients to BRAF (brafi) and MEK (MEKI) inhibitors that block the mitogen-activated protein kinase (MAPK) pathway limits their long-term use. On the other hand, immune checkpoint inhibitors can only improve the prognosis of some patients, and the molecular mechanism of lack of response is being studied. Activating MAPK pathway through BRAF mutation may be a potential molecular target to overcome melanoma cells escaping immune surveillance.

## 5. Conclusion

In our study, a systematic view of the research hotspots, future directions, and evolutionary process of NF-*κ*B in melanoma study was provided through bibliometrics analysis. It will help the younger generation of researchers who have just entered this field and experts who have been doing research in this field for a long time to better understand the current situation, trends, and future development hotspots of global research.

## Figures and Tables

**Table 1:** Publications in the 10 most productive countries/regions.

Rank	Country	Np	Nc	H-index
1	The USA	847	66218	117
2	China	507	14104	58
3	Germany	171	11693	50
4	Japan	164	14219	46
5	South Korea	135	4902	39
6	Italy	131	6006	42
7	India	97	3331	33
8	Australia	76	4153	37
9	England	75	6830	33
10	France	75	3372	32

**Table 2:** Publications in the 10 most productive affiliations.

Rank	Affiliation	Np	Nc	H-index
1	League of European Research Universities (LERU)	98	8880	40
2	University of Texas System	97	13212	47
3	University of California System	70	4283	34
4	UT MD Anderson Cancer Center	69	9518	40
5	Harvard University	52	5859	31
6	National Institutes of Health NIH USA	52	6299	34
7	Institut National de la Sante et de la Recherche Medicale (Inserm)	51	2351	27
8	US Department of Veterans Affairs	39	2261	28
9	Veterans Health Administration (VHA)	38	2261	28
10	Helmholtz Association	37	2140	24

**Table 3:** Publications in the 10 most productive authors.

Rank	Author	Affiliation	Country	NP	NC	H-index
1	Richmond A.	Vanderbilt University	The USA	29	3028	26
2	Kuttan G.	Amala Cancer Research Center	India	20	879	16
3	Ivanov V. N.	Columbia University	The USA	19	541	14
4	Li G.	University of British Columbia	Canada	16	955	14
5	Fisher P. B.	Virginia Commonwealth University	The USA	10	616	10
6	Aggarwal B. B.	University of Texas System	The USA	14	4745	13
7	Hei, T. K.	Columbia University	The USA	13	456	13
8	Kim S. H.	Kongju National University	South Korea	13	395	11
9	Sarkar, D.	Virginia Commonwealth University	The USA	13	1161	13
10	Akira, S.	Osaka University	Japan	12	5694	11

**Table 4:** Publications in the 10 most productive journals.

Rank	Journal	Np	Nc	H-index	IF (2020)
1	PloS One	62	2014	26	3.24
2	Cancer Research	59	4097	39	12.701
3	Oncogene	57	5962	37	9.867
4	Journal of Immunology	50	4168	33	5.422
5	Journal of Biological Chemistry	49	3467	32	5.157
6	Clinical Cancer Research	34	2906	26	12.531
7	International Journal of Molecular Sciences	33	855	13	5.924
8	Cancer Letters Netherlands	28	2160	19	8.679
9	Journal of Investigative Dermatology	28	966	20	8.551
10	Oncotarget	28	931	17	Removed

**Table 5:** Top 10 cocited journals.

Id	Source	Citations	Total link strength
1	Cancer Research	6796	625492
2	Journal Biological Chemistry	6555	589980
3	Nature	4323	423603
4	Proceedings of the National Academy of Sciences of the USA	4139	419771
5	Oncogene	3934	362160
6	Journal of Immunology	3894	361322
7	Cell	3357	333182
8	Science	2851	277844
9	Clinical Cancer Research	2383	227920
10	PloS One	1779	216877

## Data Availability

The data of this study are available from the corresponding author.

## References

[1] Kozovska Z., Gabrisova V., Kucerova L. (2016). Malignant melanoma: diagnosis, treatment and cancer stem cells. *Neoplasma*.

[2] Hong A. M., Waldstein C., Shivalingam B. (2021). Management of melanoma brain metastases: evidence-based clinical practice guidelines by cancer council Australia. *European Journal of Cancer*.

[3] Patel H., Yacoub N., Mishra R. (2020). Current advances in the treatment of BRAF-mutant melanoma. *Cancers*.

[4] Leonardi G. C., Falzone L., Salemi R. (2018). Cutaneous melanoma: from pathogenesis to therapy (review). *International Journal of Oncology*.

[5] Venyo A. K.-G. (2014). Melanoma of the urinary bladder: a review of the literature. *Surgery Research and Practice*.

[6] Wellbrock C., Karasarides M., Marais R. (2004). The RAF proteins take centre stage. *Nature Reviews Molecular Cell Biology*.

[7] Meier F., Schittek B., Busch S. (2005). The Ras/Raf/MEK/ERK and PI3K/AKT signaling pathways present molecular targets for the effective treatment of advanced melanoma. *Frontiers in Bioscience*.

[8] Garraway L. A., Widlund H. R., Rubin M. A. (2005). Integrative genomic analyses identify MITF as a lineage survival oncogene amplified in malignant melanoma. *Nature*.

[9] Yenmis G., Yaprak Sarac E., Besli N. (2021). Anti-cancer effect of metformin on the metastasis and invasion of primary breast cancer cells through mediating NF-*κ*B activity. *Acta Histochemica*.

[10] Zubair A., Frieri M. (2013). Role of nuclear factor-*κ*B in breast and colorectal cancer. *Current Allergy and Asthma Reports*.

[11] Zinatizadeh M. R., Schock B., Chalbatani G. M., Zarandi P. K., Jalali S. A., Miri S. R. (2021). The nuclear factor kappa B (NF-*κ*B) signaling in cancer development and immune diseases. *Genes & Diseases*.

[12] Jiao Q., Zou L., Liu P., Chi T., Cao X., Zou L. (2013). Investigation of xanthoceraside-induced apoptosis in human melanoma cancer A375 S2 cells. *Journal of Shenyang Pharmaceutical University*.

[13] Ueda Y., Richmond A. (2006). NF-kappa B activation in melanoma. *Pigment Cell Research*.

[14] Stansel T., Wickline S. A., Pan H. (2020). NF-*κ*B inhibition suppresses experimental melanoma lung metastasis. *Journal of Cancer Science and Clinical Therapeutics*.

[15] Shomali N., Marofi F., Tarzi S. (2021). HSP90 inhibitor modulates HMGA1 and HMGB2 expression along with cell viability via NF-*κ*B signaling pathways in melanoma in-vitro. *Gene Reports*.

[16] de Donatis G. M., Pape E. L., Pierron A. (2016). NF-*κ*B2 induces senescence bypass in melanoma via a direct transcriptional activation of EZH2. *Oncogene*.

[17] Chen M., Osman I., Orlow S. J. (2009). Effect of celastrol on temozolomide cytotoxicity in melanoma cells and inhibition of NF-*κ*B signaling. *Journal of Clinical Oncology*.

[18] Gallagher S. J., Mijatov B., Gunatilake D. (2014). Control of NF-*κ*B activity in human melanoma by bromodomain and extra-terminal protein inhibitor I-BET151. *Pigment Cell & Melanoma Research*.

[19] Kokol P., Blažun Vošner H., Zavrsnik J. (2021). Application of bibliometrics in medicine: a historical bibliometrics analysis. *Health Information and Libraries Journal*.

[20] Shah S. M., Ahmad T., Chen S. Z., Yuting G., Liu X. Y., Yuan Y. G. (2021). A bibliometric analysis of the one hundred most cited studies in psychosomatic research. *Psychotherapy and Psychosomatics*.

[21] Song Y. J., Ma P., Gao Y., Xiao P. G., Xu L. J., Liu H. B. (2021). A bibliometrics analysis of metformin development from 1980 to 2019. *Frontiers in Pharmacology*.

[22] Gonzalez-Alcaide G. (2021). Bibliometric studies outside the information science and library science field: uncontainable or uncontrollable?. *Scientometrics*.

[23] Luo J. L., Hu Y. D., Bai Y. H. (2021). Bibliometric analysis of the blockchain scientific evolution: 2014–2020. *IEEE Access*.

[24] Koseoglu M. A., Rahimi R., Okumus F., Liu J. (2016). Bibliometric studies in tourism. *Annals of Tourism Research*.

[25] Dong Y., Liu L., Han J. (2022). Worldwide research trends on artemisinin: a bibliometric analysis from 2000 to 2021. *Frontiers of Medicine*.

[26] Deng P., Shi H., Pan X. (2022). Worldwide research trends on diabetic foot ulcers (2004–2020): suggestions for researchers. *Journal of Diabetes Research*.

[27] Wang S., Zhou H., Zheng L. (2021). Global trends in research of macrophages associated with acute lung injury over past 10 years: a bibliometric analysis. *Frontiers in Immunology*.

[28] Chen C. M. (2006). CiteSpace II: detecting and visualizing emerging trends and transient patterns in scientific literature. *Journal of the American Society for Information Science and Technology*.

[29] Hirsch J. E. (2005). An index to quantify an individual’s scientific research output. *Proceedings of the National Academy of Sciences*.

[30] Ahn A., Rodger E. J., Motwani J. (2021). Transcriptional reprogramming and constitutive PD-L1 expression in melanoma are associated with dedifferentiation and activation of interferon and tumour necrosis factor signalling pathways. *Cancers*.

[31] Castañeda-Reyes E. D., Perea-Flores M. D J., Davila-Ortiz G., Lee Y., Gonzalez de Mejia E. (2020). Development, characterization and use of liposomes as amphipathic transporters of bioactive compounds for melanoma treatment and reduction of skin inflammation: a review. *International Journal of Nanomedicine*.

[32] Susok L., Said S., Reinert D. (2022). The pan-immune-inflammation value and systemic immune-inflammation index in advanced melanoma patients under immunotherapy. *Journal of Cancer Research and Clinical Oncology*.

[33] Neagu M., Constantin C., Caruntu C., Dumitru C., Surcel M., Zurac S. (2019). Inflammation: a key process in skin tumorigenesis. *Oncology Letters*.

[34] Voiculescu V. M., Lisievici C. V., Lupu M. (2019). Mediators of inflammation in topical therapy of skin cancers. *Mediators of Inflammation*.

[35] Tucci M., Passarelli A., Mannavola F. (2019). Immune system evasion as hallmark of melanoma progression: the role of dendritic cells. *Frontiers in Oncology*.

[36] Madonna G., Ullman C. D., Gentilcore G., Palmieri G., Ascierto P. A. (2012). NF-kappa B as potential target in the treatment of melanoma. *Journal of Translational Medicine*.

[37] Dhawan P., Su Y., Thu Y. M. (2008). The lymphotoxin-beta receptor is an upstream activator of NF-kappa B-mediated transcription in melanoma cells. *Journal of Biological Chemistry*.

[38] Xu A., Lee J., Zhao Y., Wang Y., Li X., Xu P. (2021). Potential effect of EGCG on the anti-tumor efficacy of metformin in melanoma cells. *Journal of Zhejiang University - Science B*.

[39] Huang F., Santinon F., Flores González R. E., del Rincon S. V. (2021). Melanoma plasticity: promoter of metastasis and resistance to therapy. *Frontiers in Oncology*.

